# Regional variation in the allocation of development assistance for health

**DOI:** 10.1186/1744-8603-10-8

**Published:** 2014-02-20

**Authors:** Michael Hanlon, Casey M Graves, Benjamin PC Brooks, Annie Haakenstad, Rouselle Lavado, Katherine Leach-Kemon, Joseph L Dieleman

**Affiliations:** 1 Institute for Health Metrics and Evaluation, University of Washington, 2301 5th Avenue #600, Seattle, WA 98121, USA; 2 World Bank, 1818H St NW, Washington DC 20433, USA

**Keywords:** Development assistance for health, Foreign aid for health, Disability-adjusted life years

## Abstract

**Background:**

The Global Burden of Disease (GBD) 2010 Study has published disability-adjusted life year (DALY) data at both regional and country levels from 1990 to 2010. Concurrently, the Institute for Health Metrics and Evaluation (IHME) has published estimates of development assistance for health (DAH) at the country-disease level for this same period of time.

**Findings:**

We use disease burden data from the GBD 2010 study and financing data from IHME to calculate ratios of DAH to DALYs across regions and diseases. We examine the magnitude of these ratios and how they have varied over time. We hypothesize that the variation in this ratio across regions would be relatively small. However, from 2006 to 2010, we find there was considerable variation in the levels of DAH per DALY across regions. For total funding, the relative standard deviation (standard deviation as a percentage of the mean) across regions was 50%. For DAH specific to HIV/AIDS, malaria and tuberculosis, the relative standard deviations were 50%, 200% and 60%, respectively. While these deviations are high, with the exception of malaria, they have decreased since the 1990s.

**Conclusions:**

There are no evident explanations for so much variation in funding across regions, especially holding the purpose of the funding constant. This suggests donors’ allocation processes have not been particularly sensitive to disease burdens. To maximize health gains, donors should explicitly incorporate new disease burden data along with the relative costs and efficacy of interventions into their allocation process.

## Findings

There is remarkable variation in the level of development assistance for health (DAH) per disability adjusted life-year (DALY) across regions and diseases. From 2006 to 2010, the rate of total DAH per all-cause DALY ranged from $16 to $78 across regions (all financial values in 2010 US dollars). The regional variation in disease-specific funding was generally more extreme. DAH per DALY ranged from $174 to $749 for HIV/AIDS, $62 to $36,030 for malaria and $38 to $305 for tuberculosis (TB). Some variation across diseases may be justified by differences in the relative costs and efficaciousness of interventions. Yet little evidence exists to explain the massive variation in disease-specific funding that we observe across regions.

## Methods

DAH is comprised of funds that originate in developed countries and are disbursed to developing countries with the stated purpose of improving population health. DAH estimates were obtained from the *Financing Global Health* series produced by the Institute for Health Metrics and Evaluation (IHME) [1-4]. To generate the 2012 report, IHME collected data from entities that contributed to DAH from 1990 to 2012. Annual reports, publicly available data, and information acquired via correspondence feed into the DAH dataset. Some data are verified through conversations with the respective organizations. We used the publicly-available dataset from the 2012 report to generate disbursement amounts through 2010, which is the most recent year for which disease-specific estimates are available [4]. In 2010, 79% of total DAH could be tracked to a specific focus area, such as HIV/AIDS, malaria or TB. Insufficient data exist on the remaining 21% to comment on its allocation across diseases or regions, but this “unallocable” fraction has decreased steadily in recent years as larger donors have embraced the practice of publicly sharing project-level data.

Disease burden is measured by a country’s number of DALYs. This metric combines the number of years of life lost due to premature mortality and the years lived with disability. It simultaneously accounts for conditions which result in mortality and those which result in disability. The metric was specifically developed to assess the burden of disease [5] and it has been adopted by the World Health Organization as its preferred metric for quantifying burden [6]. Total DALYs are calculated by summing disease-specific DALYs, such as those directly attributable to HIV/AIDS, malaria and TB, among many other conditions. For further description, see *The Lancet*’s issue devoted to the Global Burden of Disease (GBD) 2010 Study [7-9].

The GBD 2010 Study produced DALY estimates for five points in time: 1990, 1995, 2000, 2005 and 2010. To pair DALYs with DAH, we sum DAH in a five-year interval such that the final year of each interval matches a DALY estimate. We sum DAH and DALYs at the regional level, with regions defined by the World Bank. We calculate regional-level ratios of DAH to DALYs. These ratios are used to generate a relative standard deviation (RSD), which is the coefficient of variation expressed as a percentage (the ratio of the standard deviation to the mean, times one hundred). RSDs are calculated for total DAH and all-cause DALYs, as well as those specific to HIV/AIDS, malaria and TB.

## Results

**Figure 1:**
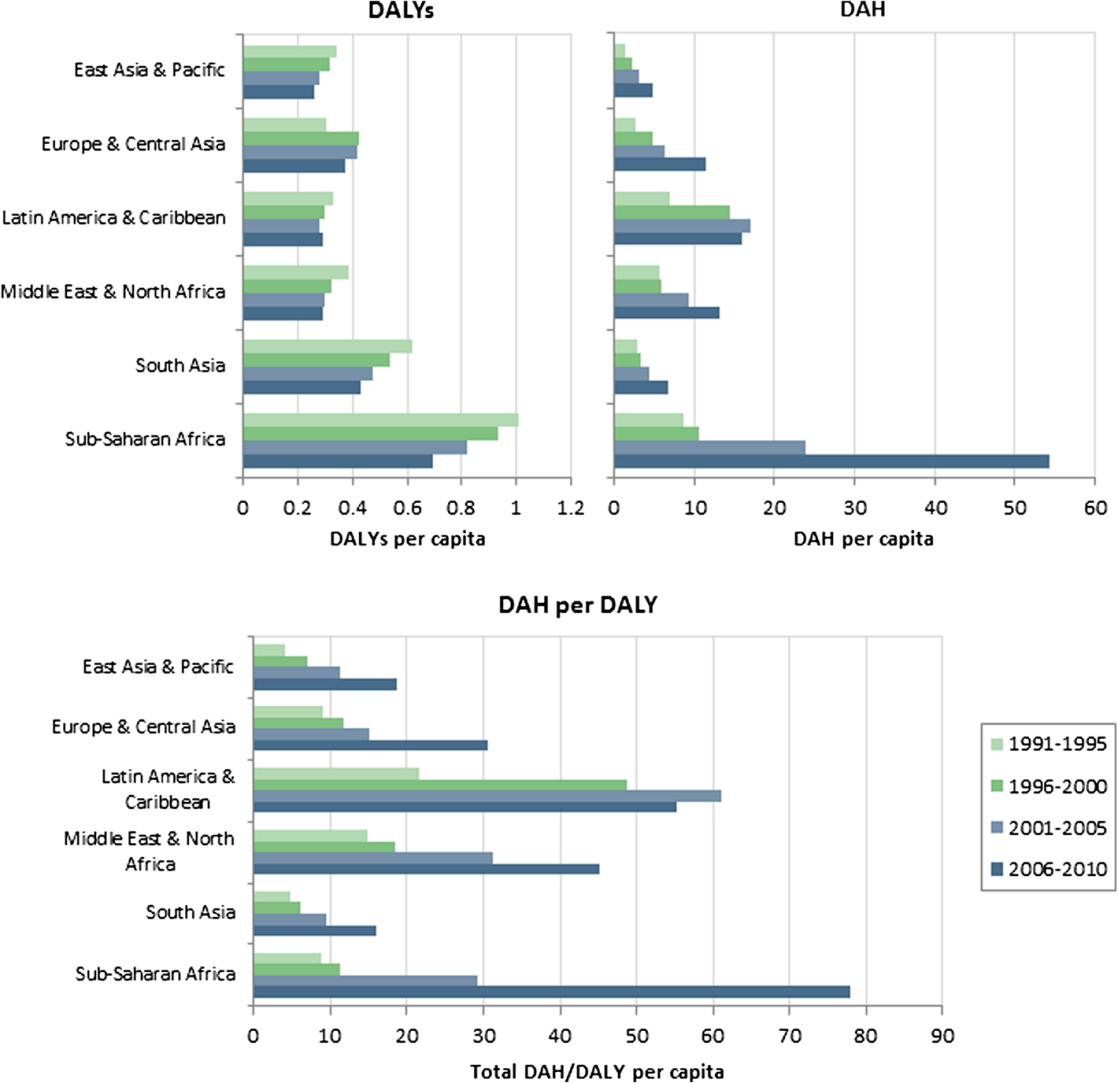
Total DAH per DALY, by region.

**Figure 2:**
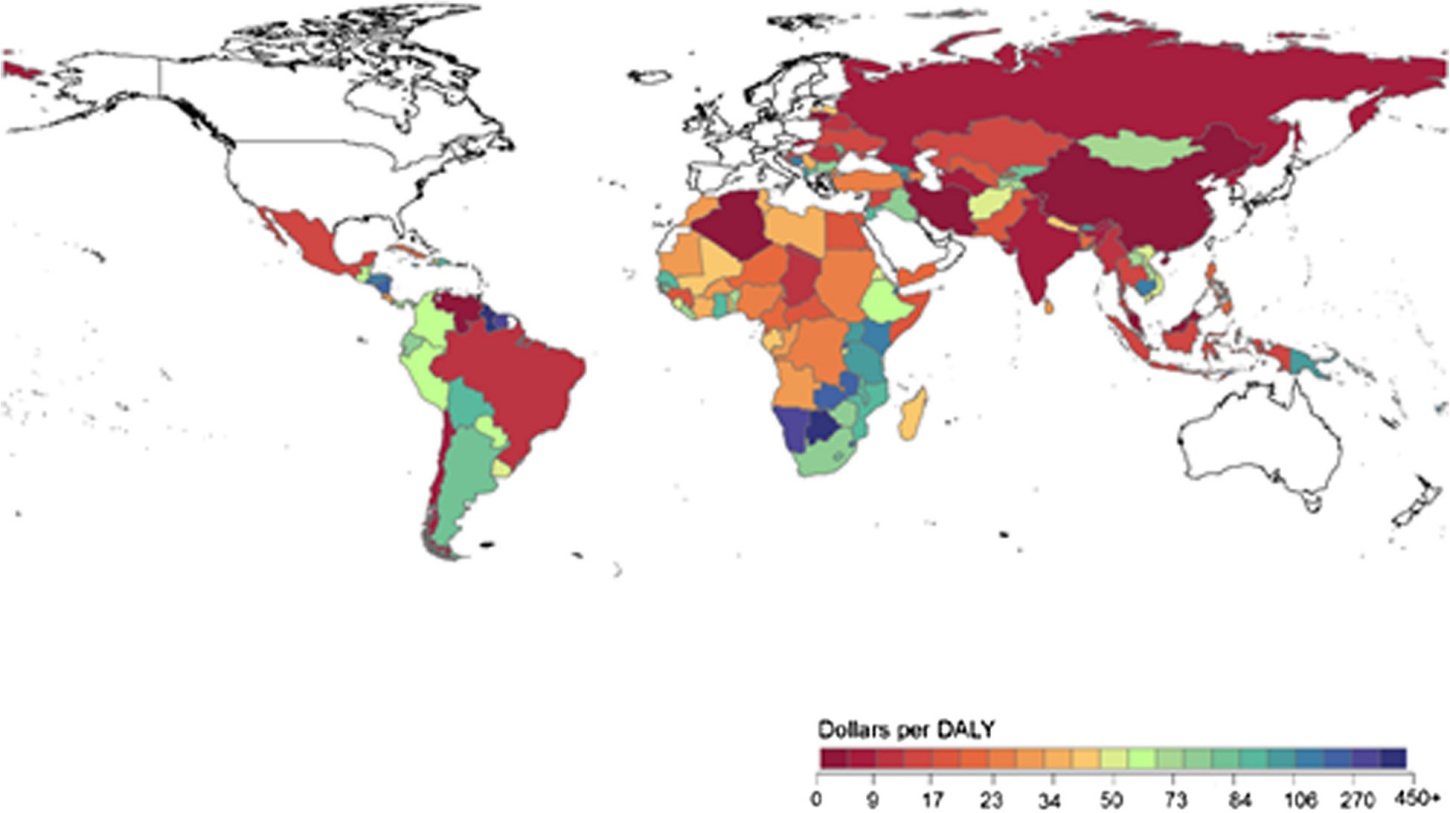
**Global map of DAH per DALY at the country level, 2006 – 2010.** DAH was aggregated at the country level from 2006 to 2010 and compared to country-level DALYs reported in the year 2010.

**Figure 3:**
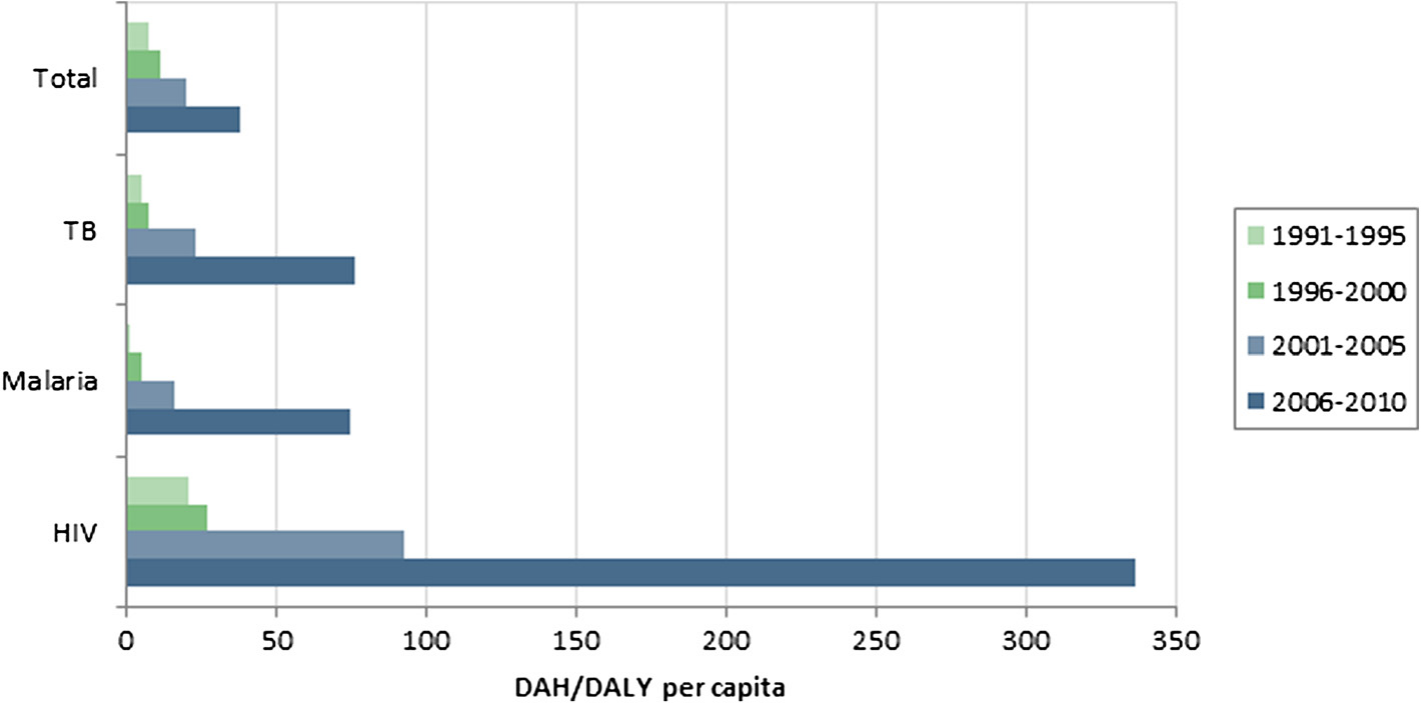
Global DAH per DALY, by disease.

Figure 1 illustrates that DALYs have been flat or decreasing over time while levels of DAH have increased dramatically since the 1990s. Consequently, the ratio of DAH to DALYs increased in all regions of the world over the past two decades. In addition to the temporal variation, there is considerable variation in the ratio across regions. South Asia, East Asia and the Pacific receive relatively little DAH per DALY, while Sub-Saharan Africa receives the most. Figure 2 illustrates DAH per DALY at the country level. It highlights country-level variation exists within regions. Figure 3 illustrates the variation across disease-specific funding. It highlights that donors provide much more DAH per HIV DALY than from other burdens.

**Figure 4:**
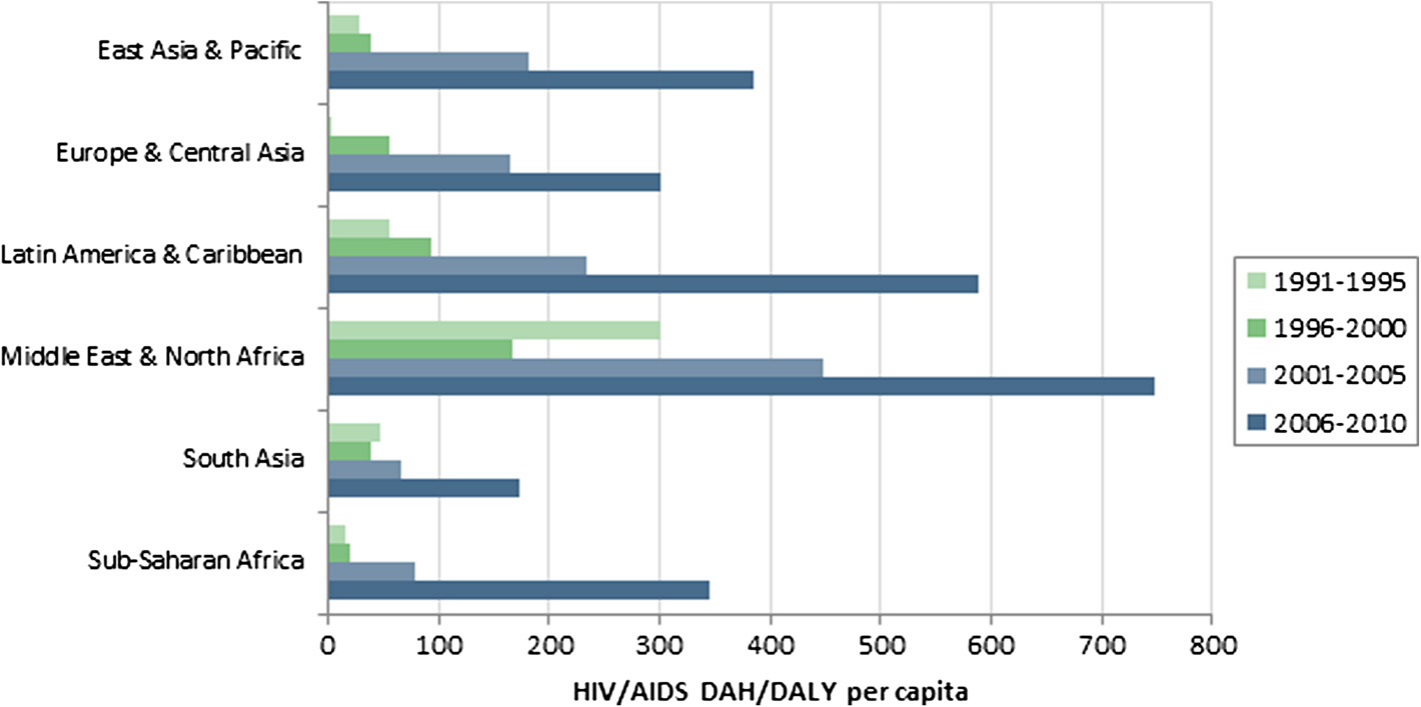
HIV/AIDS DAH per HIV/AIDS DALY, by region.

**Figure 5:**
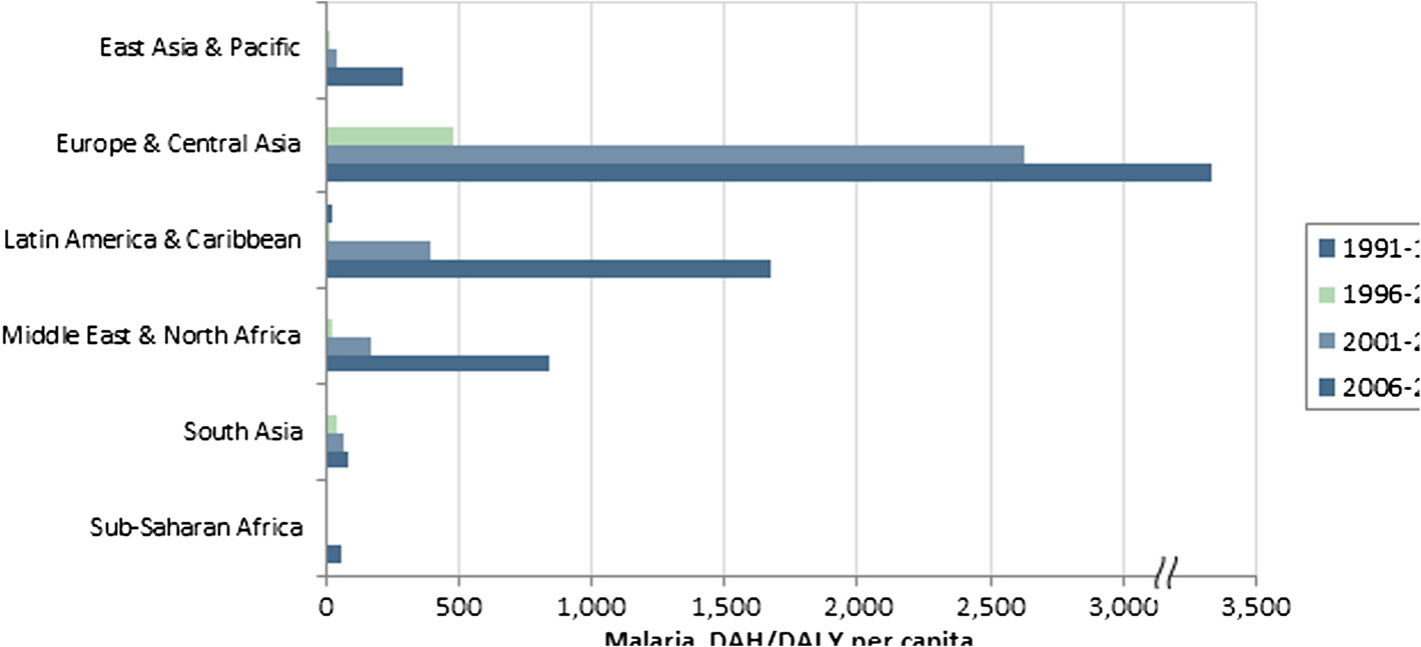
Malaria DAH per malaria DALY, by region.

**Figure 6:**
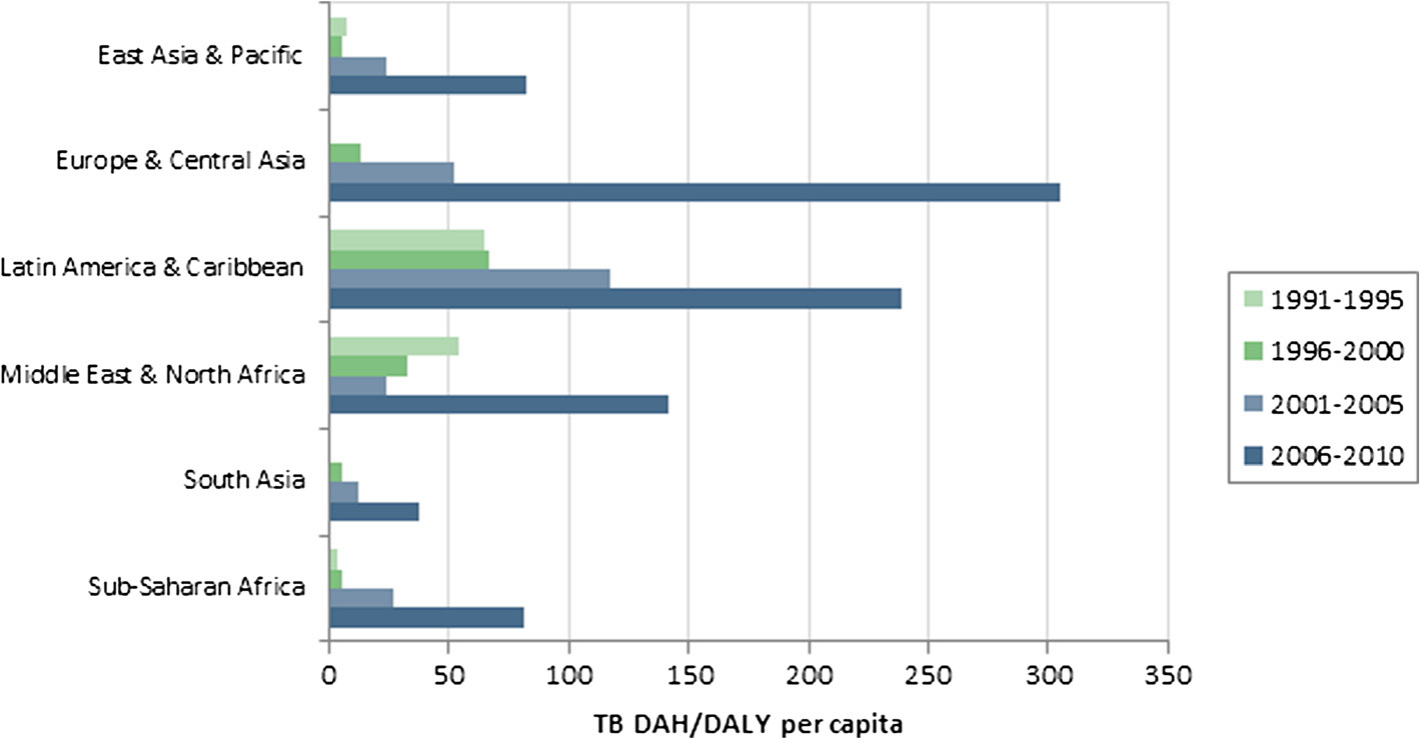
TB DAH per TB DALY, by region.

Figures 4, 5 and 6 illustrate regional levels of DAH per DALY for HIV/AIDS, malaria and TB, respectively. For HIV/AIDS, the Middle East and Latin America receive high levels of funding relative to the burden. South Asia receives low levels relative to the burden. For malaria, extremely high levels are allocated to Europe and Central Asia, in large part because of the relatively low burden in that region. For TB, high levels are allocated to Europe and Central Asia and Latin America, while low levels are allocated to Sub-Saharan Africa and South Asia. Table 1 reports the RSD in funding over time. While large variations exist from 2006 to 2010, in general the level of variation has actually decreased over time. The notable exception is funding for malaria, in which the variation continues to increase because of increasingly large allocations of DAH to countries in Europe and Central Asia.

**Table 1:** Relative standard deviations in funding across regions over time

**Time period**	**Funding area**			
	**Total**	**HIV/AIDS**	**Malaria**	**TB**
1991 – 1995	58%	124%	145%	125%
1996 – 2000	86%	72%	179%	105%
2001 – 2005	68%	65%	170%	84%
2006 – 2010	53%	45%	203%	64%

## Discussion

A large literature examines how factors such as per capita income, population levels and measures of governance affect foreign aid allocations [10-24]. Some of this literature explicitly focuses on disease burden [23,24]. The practicality of this literature is limited because many studies focus on the associations between variables, rather than causal pathways. Regardless, to our knowledge no study has focused on the ratio of assistance to burden. We believe this is a useful metric that should influence donor behavior.

We find massive variation in this ratio, which suggests donors have not been sensitive to disease burdens in making their allocations. This finding may be somewhat expected, for four reasons. First, some large donors like the Global Fund have employed allocation mechanisms in which grants are functions of many factors in addition to burden [25]. Second, some organizations may avoid allocating aid based on burden averted to avoid the perverse dynamic of punishing recipients who successfully lower the burden from a specific disease. Third, for some donors, the true purpose of health aid may extend beyond improving population health. Finally, historically data on burden have not been readily accessible. Even if donors wanted to focus on burden, it was in some cases operationally impractical.

Variation in all-cause funding across regions is in part due to donors’ demand to target funding to several high-profile diseases, as illustrated in Figure 3. For example, populations in Sub-Saharan Africa suffer disproportionately from HIV/AIDS. As donors prioritize that disease, countries in Sub-Saharan Africa will receive disproportionately large allocations of DAH, which serves to increase the variation in DAH to DALYs across countries. The variation across diseases may in part be justified by differences in the relative costs and effectiveness of disease-specific interventions. However, given the current evidence about costs and effectiveness, it is unclear that those factors alone could explain the observed differences. The variation across regions for a given type of funding, illustrated in Figures 4,5 and 6, is a more surprising and somewhat inexplicable result. Latin America and the Caribbean receive consistently high allocations across diseases, while South Asia’s allocations are consistently low. However, other region-disease combinations are highly idiosyncratic.

As donors allocate capital in the future, and in some cases reconsider their allocation strategies, we believe three policy issues are relevant to address. First, to what degree does population health matter in the allocation of health aid? Second, to the degree that burden influences the allocation, are donors using consistent measures of burden across diseases? Third, holding the decision to address burden from a specific cause, are the allocations equitable across regions? DALYs from the Global Burden of Disease 2010 Study are a useful tool to address the second and third issues. This data could serve as a useful input to donors’ allocation processes and reduce variations in the future.

## Competing interests

The authors declare that they have no competing interests.

## Authors’ contributions

MH was the principal author of the manuscript and managed the research team that completed the project. CG and BB were the principal analysts. AK and RL provided support and feedback on the analysis and on the interpretation of the results. Note that RL contributed to this study while she served as a Post-Graduate Fellow at IHME. KLK and JD contributed to writing the manuscript, and JD contributed to the management of the scientific process. All authors read and approved the final manuscript.
